# Dysregulation of MicroRNA-34a Expression in Head and Neck Squamous Cell Carcinoma Promotes Tumor Growth and Tumor Angiogenesis

**DOI:** 10.1371/journal.pone.0037601

**Published:** 2012-05-22

**Authors:** Bhavna Kumar, Arti Yadav, James Lang, Theodoros N. Teknos, Pawan Kumar

**Affiliations:** 1 Department of Otolaryngology-Head and Neck Surgery, The Ohio State University, Columbus, Ohio, United States of America; 2 The Ohio State University Comprehensive Cancer Center, The Ohio State University, Columbus, Ohio, United States of America; Enzo Life Sciences, Inc., United States of America

## Abstract

**Background:**

MicroRNAs (miRs) are small non-coding RNAs that play an important role in cancer development where they can act as oncogenes or as tumor-suppressors. miR-34a is a tumor-suppressor that is frequently downregulated in a number of tumor types. However, little is known about the role of miR-34a in head and neck squamous cell carcinoma (HNSCC).

**Methods and Results:**

miR-34a expression in tumor samples, HNSCC cell lines and endothelial cells was examined by real time PCR. Lipofectamine-2000 was used to transfect miR-34a in HNSCC cell lines and human endothelial cells. Cell-proliferation, migration and clonogenic survival was examined by MTT, Xcelligence system, scratch assay and colony formation assay. miR-34a effect on tumor growth and tumor angiogenesis was examined by *in vivo* SCID mouse xenograft model. Our results demonstrate that miR-34a is significantly downregulated in HNSCC tumors and cell lines. Ectopic expression of miR-34a in HNSCC cell lines significantly inhibited tumor cell proliferation, colony formation and migration. miR-34a overexpression also markedly downregulated E2F3 and survivin levels. Rescue experiments using microRNA resistant E2F3 isoforms suggest that miR-34a-mediated inhibition of cell proliferation and colony formation is predominantly mediated by E2F3a isoform. In addition, tumor samples from HNSCC patients showed an inverse relationship between miR-34a and survivin as well as miR-34a and E2F3 levels. Overexpression of E2F3a completely rescued survivin expression in miR-34a expressing cells, thereby suggesting that miR-34a may be regulating survivin expression via E2F3a. Ectopic expression of miR-34a also significantly inhibited tumor growth and tumor angiogenesis in a SCID mouse xenograft model. Interestingly, miR-34a inhibited tumor angiogenesis by blocking VEGF production by tumor cells as well as directly inhibiting endothelial cell functions.

**Conclusions:**

Taken together, these findings suggest that dysregulation of miR-34a expression is common in HNSCC and modulation of miR34a activity might represent a novel therapeutic strategy for the treatment of HNSCC.

## Introduction

Head and neck squamous cell carcinoma (HNSCC), which includes cancers of oral cavity, oropharynx, larynx, and hypopharynx accounts for approximately 600,000 new cases every year and is sixth leading cancer by incidence worldwide [1]. The most important risk factors identified so far are tobacco use and alcohol consumption, which seem to have a synergistic effect [2]. In recent years, the incidence of oropharyngeal cancers, particularly in the western world, has markedly increased, which may be related to increase in oral and oropharyngeal human papillomavirus (HPV) infections [3]. Despite advancements in surgical and other therapeutic regimens, 5 year survival rates for head and neck patients have stayed around 50% during the last two decades [4]. Patients that survive with surgery and/or chemo-radiation treatment (CRT) often live with significant cosmetic and functional defects. The limited information available on the molecular carcinogenesis of HNSCC and the genetic and biological heterogeneity of the disease has hampered the development of novel therapeutic strategies.

Cancer is a complex genetic disease in which deregulated cell growth arises due to defects in major pathways that are fundamental for normal homeostasis [1]. Evidence is emerging that alterations in the expression of microRNAs (miRs) may play a key role in cancer development and progression [5]. MicroRNAs (miRs), first described about 18 years ago in *Caenorhabditis elegans* [6], are small non-coding RNAs that act in concert to regulate expression of myriad target Proteins. Today more than 17000 microRNA’s in over 153 species have been identified (miRBase Sequence Database - Release 17; www.miRbase.org), of which around 1400 are in humans. Most miRs are evolutionarily conserved and often found in clusters [7]. In mammals, mature miRs are recognized by specific proteins to form RNA-inducing silencing complex (RISC) and associate with 3′-untranslated regions (3′-UTR) of specific target messenger RNA (mRNA) to suppress translation or occasionally induce their degradation [5]. Changes in miR expression profile have been shown to be associated with a variety of human cancers [8], [9], [10], [11]. More importantly, it has been found that they are differentially expressed in tumor tissues compared to noncancerous tissues [12], [13], [14], [15], [16]. miRs can function as tumor suppressors or oncogenes, depending on whether they specifically target oncogenes or tumor suppressor genes. Therefore, understanding the molecular mechanisms by which these miRs play a role in deregulated cellular signaling in the head and neck cancer cell might help develop better therapeutic strategies for treatment of this disease.

Recent studies have highlighted the role of miR-34a as a tumor suppressor in a number of tumor types including prostate cancer, hepatocellular carcinoma, neuroblastoma and colon cancer [17], [18], [19], [20], [21], [22]. miR-34a was originally discovered as a potential tumor suppressor that is downregulated and induces apoptosis in neuroblastoma cells [20]. Subsequently, it was shown to be a transcriptional target of p53 protein [23], [24]. Functionally miR-34a was found to affect tumor cell proliferation, apoptosis, senescence, invasion, metastasis and drug resistance [21], [22], [23], [25], [26], [27]. However, very little is known about the role and expression status of miR-34a in head and neck cancers. In this study, we examined if loss of miR-34a in head and neck cancers promotes tumor growth and tumor angiogenesis. Our results demonstrate that miR-34a expression is significantly downregulated in primary tumors from head and neck cancer patients as well as in head and neck cancer cell lines. Ectopic expression of miR-34a in head and neck cell lines significantly inhibited tumor cell proliferation, migration and colony formation by downregulating the expression of E2F3 and survivin. In addition, miR-34a significantly inhibited tumor growth and tumor angiogenesis in a SCID mouse xenograft model. Interestingly, miR-34a inhibited tumor angiogenesis by blocking VEGF secretion by tumor cells as well as directly inhibiting endothelial cell functions.

## Results

### miR-34a Expression is Significantly Downregulated in Primary Tumor Samples and Tumor Cell Lines from the Head and Neck Cancer Patients

**Figure 1:**
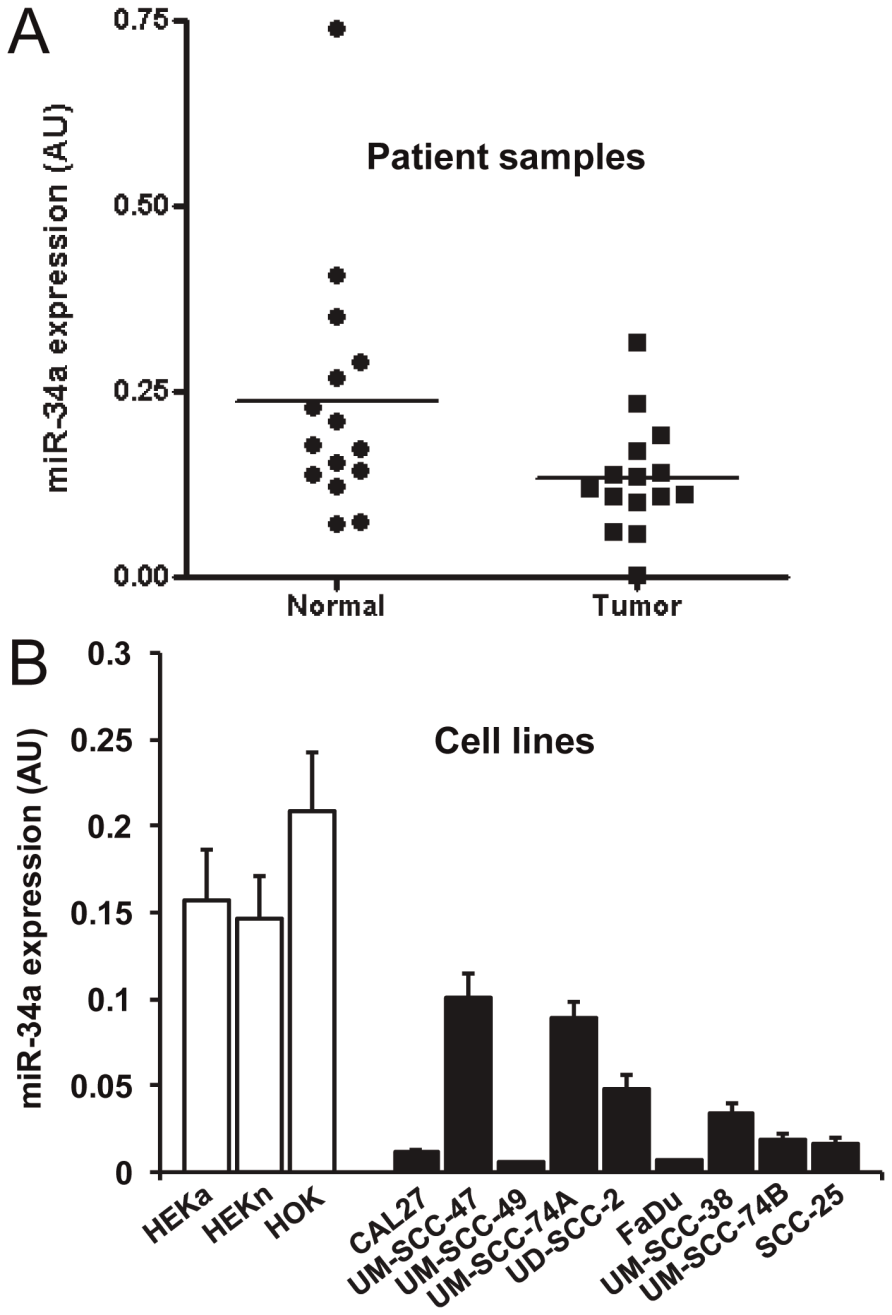
Expression of miR-34a is downregulated in HNSCC cell lines and patient’s tumor samples. *A:* miR-34a expression was examined in primary tumor samples (n = 15) or in adjacent normal mucosa (n = 15) of head and neck cancer patients by quantitative real time PCR (RT-PCR) and statistical significance was analyzed by Mann-Whitney test (p value 0.022). *B:* miR-34a expression was analyzed in 9 head and neck cancer cell lines by RT-PCR and compared with 3 normal human keratinocyes [human oral keratinocytes (HOK); human epidermal keratinocytes, adult (HEKa) and human epidermal keratinocytes, neonatal (HEKn)].

We measured miR-34a expression levels in 30 frozen samples from head and neck cancer patients (15 tumors and 15 adjacent normal controls) by TaqMan real time RT-PCR. Out of 15 tumor samples, 6 were from oral cavity, 5 from oropharynx and 4 from larynx. Our results show that miR-34a expression is significantly decreased (Mann-Whitney test; p value 0.0226) in head and neck tumors as compared to adjacent normal tissue (Figure 1A). We did not observe any significant differences in miR-34a levels in tumor samples from the different head and neck sub-sites. We next examined if head and neck tumor cell lines also exhibit similar downregulated expression of miR-34a as compared to normal keratinocyes. We examined miR-34a levels in 3 different normal keratinocyte cell types [human oral keratinocytes (HOK); human epidermal keratinocytes, adult (HEKa) and human epidermal keratinocytes, neonatal (HEKn)] and 9 head and neck cancer cell lines. Indeed, miR-34a expression was markedly downregulated in all the head and neck cancer cell lines that were examined (Figure 1B).

### miR-34a Inhibits Head and Neck Tumor Cell Proliferation, Colony Formation and Migration

**Figure 2:**
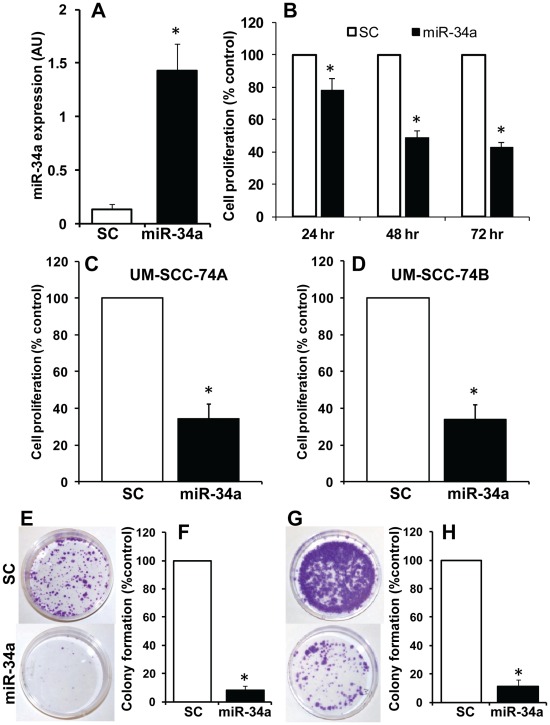
Ectopic expression of miR-34a inhibits tumor cell proliferation and colony formation. *A:* Tumor cells (UM-SCC-74A) were transfected with miR-34a or scrambled control microRNAs (SC) and miR-34a expression was analyzed by quantitative real time PCR (RT-PCR). *, represent a significant difference (p<0.05). *B:* Cell proliferation was measured by MTT assay at different time points. Percentage cell proliferation for tumor cells transfected with miR-34a was calculated by adjusting proliferation index of tumor cells transfected with SC to 100. *, represent a significant inhibition (p<0.05) of cell proliferation in miR-34a expressing tumor cells as compared to SC. *C-D:* Cell proliferation was measured using Xcelligence system using the RTCA DP instrument. Percentage cell proliferation for tumor cells transfected with ectopic miR-34a was calculated by adjusting proliferation index of tumor cells transfected with SC to 100. *, represent a significant inhibition (p<0.05) of cell proliferation in miR-34a expressing tumor cells at 48 hrs as compared to SC. *E-H:* Effect of miR-34a on tumor cell colony formation (E-F; UM-SCC-74A and G-H; UM-SCC-74B) was examined by culturing tumor cells (5,000) in 60 mm Petri dishes for 14 days. Colony numbers in each assay was quantified by Alpha Innotech (San Leandro, CA) imaging software and percentage colony formation for tumor cells transfected with miR-34a was calculated by adjusting tumor cells transfected with SC to 100. *, represent a significant inhibition (p<0.05) of tumor cell colony formation in miR-34a expressing tumor cells as compared to SC.

**Figure 3:**
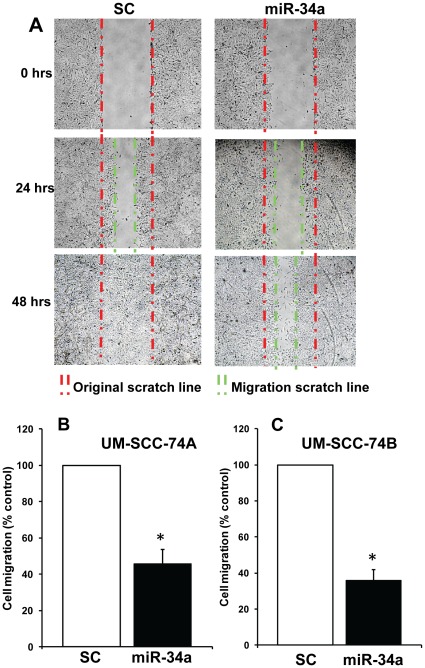
miR-34a significantly inhibits tumor cell migration. *A:* Tumor cell (UM-SCC-74A) motility was examined by scratch assay. Red dotted lines mark the edges at the start of experiments, and green dotted lines mark the edges at the end of experiments. *B-C:* Tumor cell migration was analyzed by Xcelligence system. Percentage cell migration for cells ectopically expressing miR-34 was calculated by adjusting migration index for tumor cells transduced with SC to 100%. *, represent a significant inhibition (p<0.05) of cell migration in miR-34a expressing tumor cells as compared to SC.

Next, we examined the role of miR-34a on head and neck tumor cell proliferation, colony formation and migration. 50 nM of pre-miR-34a or scrambled control RNA was used to transfect head and neck cancer cell lines. Ectopic expression of miR-34a in transfected cells (UM-SCC-74A) was confirmed by RT-PCR (Figure 2A). Similar ectopic expression of miR-34a was observed in UM-SCC-74B cells (data not shown). We performed parallel experiments for cell proliferation using Xcelligence system and MTT assay (Roche Diagnostics, Indianapolis, IN). Ectopic expression of miR-34a significantly inhibited cell proliferation in UM-SCC-74A cells (MTT assay; Figure 2B & Figure S2 and Xcelligence assay; Figure 2C) and UM-SCC-74B cells (Figure 2D). Similarly, miR-34a transfection in both the head and neck cancer cell lines markedly decreased tumor cell colony formation (Figure 2E-H) and tumor cell migration (Scratch assay; Figure 3A and Xcelligence migration assay; Figure 3B-C). Cell migration by Xcelligence was measured at 24 hrs and there was 58% and 63% cell migration inhibition in UMSCC-74A and UM-SCC-74B cells, respectively (Figure 3B-C). Whereas only 20% cell proliferation inhibition (Figure 2) was observed at the same time point (24 hrs), thereby suggesting that miR-34a predominantly affected cell migration at 24 hrs.

### miR-34a Inhibits Tumor Cell Proliferation and Colony Formation by Downregulating E2F3 and Survivin

**Figure 4:**
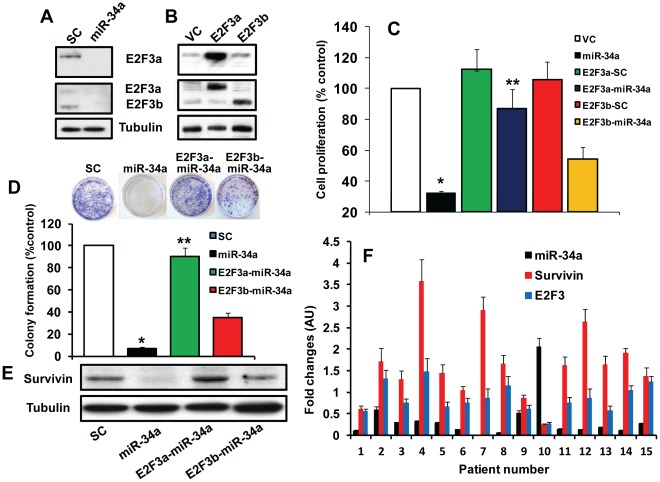
miR-34a mediates its biological function by downregulating E2F3 and survivin levels. *A:* UM-SCC-74A cells were transfected with miR-34a or scrambled control (SC). Seventy two hrs after transfection whole cell lysate was prepared and Western blotted using antibody specific for E2F3a (top panel) or antibody that binds both E2F3a and E2F3b (middle panel). Equal protein loading in Western blotting was verified by stripping the blots and reprobing with tubulin antibody. *B:* MicroRNA resistant E2F3a or E2F3b were stably overexpressed in UM-SCC-74A cells by retroviral vector and E2F3a and E2F3b overexpression was verified by Western blot analysis. *C:* UM-SCC-74A cells overexpressing E2F3a, E2F3b or vector alone (VC) were ectopically transfected with miR-34a or scrambled control (SC). After 72 hrs, cell proliferation was measured using Xcelligence system. *D:* Tumor cell colony formation was examined by culturing tumor cells (5,000) in 60 mm Petri dishes for 14 days and colony numbers is each assay was quantified by Alpha Innotech (San Leandro, CA) imaging software. *E:* UM-SCC-74A cells overexpressing E2F3a, E2F3b or vector alone (VC) were ectopically transfected with miR-34a or SC. After 72 hrs, whole cell lysates were Western blotted for survivin. Equal protein loading in Western blotting was verified by stripping the blots and reprobing with tubulin antibody. *F:* miR-34a, survivin and E2F3 mRNA expression from primary tumors of 15 head and neck cancer patients was analyzed by real time RT-PCR.

We next sought to identify the target mRNAs of miR-34a that regulate head and neck tumor cell function. TargetScan data base (www.targetscan.org) search revealed several growth regulatory mRNAs that contains conserved miR-34a recognition sites in their 3′-UTR. In this study, we focused our attention to E2F3a and E2F3b because E2F3 family of transcription factors play an important role in cell cycle regulation, cell proliferation and differentiation [28], [29], [30]. To confirm that miR-34a regulates the endogenous expression of E2F3a and E2F3b, we ectopically introduced miR-34a in UM-SCC-74A cells and examined E2F3a/b levels in these cells 72 hrs post transfection. We used two different antibodies against E2F3, including a monoclonal antibody that specifically recognizes the unique N-terminal portion of E2F3a (Figure 4A-B, top panel) and this antibody is referred as E2F3a. We also used a polyclonal antibody that recognizes a region close to C terminus, which is common to both E2F3a and E2F3b [29] (Figure 4A-B, middle panel). Expression of both E2F3a and E2F3b proteins was markedly downregulated in miR-34a transfected cells as compared to cells transfected with scrambled control RNA (Figure 4A).

As both E2F3 isoforms were downregulated in miR-34a transfected cells, we performed isoform-specific rescue experiments to understand the specific contribution of E2F3a and E2F3b in miR-34a mediated tumor suppressor function. We used microRNA resistant constructs for E2F3a and E2F3b [29]. These constructs were generated by modifying the microRNA targeting sequence by introducing three silent base changes (a gift from Dr. David Dynlacht, New York University). Expression of E2F3a and E2F3b in UM-SCC-74A cells showed significantly elevated protein levels as compared to cells transfected with control vector (VC) (Figure 4B). Overexpression of E2F3a or E2F3b in UM-SCC-74A cells showed a modest increase in cell proliferation (12% and 6% respectively; Figure 4C). Interestingly, E2F3a overexpression in UM-SCC-74A cells significantly rescued tumor cells from miR-34a-mediated inhibition of cell proliferation (56%, Figure 4C) and colony formation (86%, Figure 4D), whereas E2F3b was only partially effective in reversing miR-34a-mediated inhibition of cell proliferation (19%, Figure 4C) and colony formation (26%, Figure 4D).

Survivin, encoded by the gene *BIRC5* is a member of the inhibitor of apoptosis proteins (IAP) family of molecules [31]. Survivin is aberrantly expressed in many malignancies including HNSCC [32] and has been shown to play a role in cancer progression and resistance to therapy [33]. Recently, miR-34a was shown to decrease survivin promoter activity [34]. In addition, survivin promoter activity is also regulated by E2F3, a key miR-34a target protein [35]. Since ectopic expression of miR34a inhibits E2F3 expression, we further examined if survivin expression is regulated by miR-34a via E2F3. Indeed, miR-34a transfection in UM-SCC-74A cells significant decreased survivin protein levels (Figure 4E). We next examined if overexpression of miR resistant E2F3a or E2F3b could rescue survivin expression in miR-34a treated cells. Overexpression of E2F3a was able to completely rescue survivin expression in miR-34a transfected UM-SCC-74A cells, whereas E2F3b was only partially effective (Figure 4E). To further validate the cell line findings of inverse relationship between miR-34a and its target proteins survivin and E2F3, we examined miR-34a, survivin and E2F3 mRNA levels in the primary head and neck tumor samples. All 15 tumor samples showed an inverse relationship between miR-34a levels and survivin (Pearson r value 0.89; p value 0.0001). 14/15 patient samples showed low levels of miR-34a and high levels of survivin mRNA. One sample (Patient # 10) showed high miR-34a and low survivin levels (Figure 4F). Similarly, miR-34a levels were inversely correlated with E2F3 levels (Pearson r value 0.81; p value 0.0002).

### miR-34a Inhibits Tumor Growth, *in vivo*

**Figure 5:**
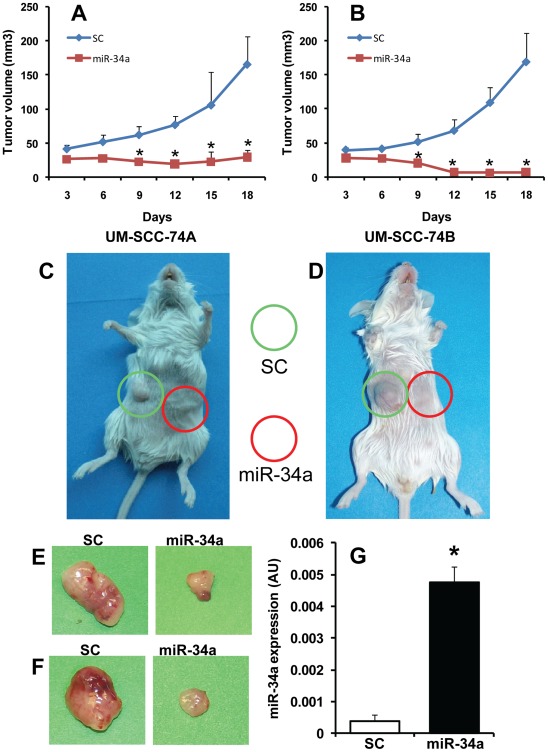
miR-34a inhibits tumor growth in vivo. Tumor cells transfected with miR-34a or SC were mixed with 100 µl of Matrigel and injected subcutaneously in the left and right flanks of SCID mice respectively (n = 5). Tumor volume measurements began on day 3 and continued twice a week until the end of the study. The length and width of the tumors were measured using a digital caliper and tumor volumes were calculated using the formula, volume (mm^3^) = L×W^2^/2 (length L, mm; width W, mm). After 18 days, tumors samples were carefully removed. *A-B:* Tumor growth curves for UM-SCC-74A and UM-SCC-74B respectively. *, represent a significant inhibition (p<0.05) of tumor growth in miR-34a group cells as compared to SC. *C-D:* Representative photographs of mice bearing UM-SCC-74A or UM-SCC-74B tumors. Green and red circles are used to highlights tumor cells transfected with scrambled control (SC) or miR-34a, respectively. *E-F:* Representative photographs for UM-SCC-74A and UM-SCC-74B tumors at day 18, respectively. *G:* miR-34a levels in UM-SCC-74A tumors at the end of the *in vivo* experiments. *, represent a significant difference (p<0.05).

To confirm the anti-tumor effects on miR-34a *in vivo*, we implanted two head and neck cell lines either expressing miR-34a or scrambled control in the right or left flanks of SCID mice, respectively. Representative photographs of SCID mice bearing tumors at day 18 and dissected tumors are shown in Figure 5C-F. Ectopic expression of miR-34a expression in both the tumor cell lines significantly inhibited tumor growth in SCID mice (Figure 5). In order to confirm that ectopic expression of miR-34a stayed throughout the *in vivo* experiments, we isolated RNA from UM-SCC-74A tumor samples at the end of study and performed real time RT-PCR. Indeed, ectopic expression of miR-34a was maintained in the tumor cells throughout the study (Figure 5G).

### miR-34a Inhibits Tumor Angiogenesis by Downregulating VEGF Secretion from Tumor Cells

**Figure 6:**
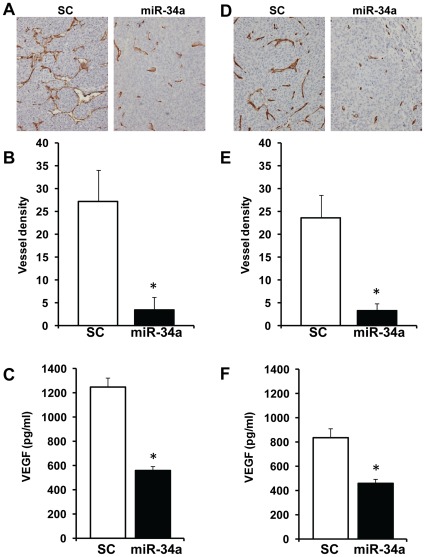
miR-34a inhibits tumor angiogenesis by downregulating VEGF secretion from tumor cells. *A-B:* Paraffin embedded tumor samples were stained with anti-CD31 antibody and microvessel density was calculated by counting 5 random high power fields (200x). *A:* Representative photographs of UM-SCC-74A tumors expressing scrambled control microRNAs (SC) or miR-34a. *B:* Microvessel density in tumor samples from scrambled control microRNAs (SC) or miR-34a groups. *, represent a significant inhibition (p<0.05) of tumor angiogenesis in miR-34a group as compared to SC. *C:* VEGF levels (pg/ml per 10^6^ tumor cells) in culture supernatants from UM-SCC-74A cells transfected with miR-34a or scrambled control microRNA (SC). *D:* Representative photographs of UM-SCC-74B tumors expressing scrambled control microRNAs (SC) or miR-34a. *E:* Microvessel density in tumor samples from scrambled control microRNAs (SC) or miR-34a groups. *F:* VEGF levels (pg/ml per 10^6^ tumor cells) in culture supernatants from UM-SCC-74B cells transfected with miR-34a or scrambled control microRNA (SC).

In our *in vivo* tumor growth study, in addition to smaller tumor size, we also observed that tumors were visually less vascular in miR-34a transduced group. VEGF is a key angiogenic protein and recent studies have shown that a number of miR-34a target proteins including E2F3, Myc and c-met can regulate VEGF expression [36], [37], [38]. Therefore, we next examined if ectopic expression of miR-34a affected tumor angiogenesis. UM-SCC-74A tumors expressing miR-34a showed a significant decrease in blood vessel density (83%) as compared to tumors expressing scrambled control RNA (Figure 6A-B). Similarly, UM-SCC-74B tumors showed 87% decrease in tumor blood vessel density (Figure 6D-E). We next examined if miR-34a inhibits tumor angiogenesis by blocking VEGF production by the tumor cells. miR-34a was ectopically expressed in tumor cells and VEGF (VEGF A) levels in the culture supernatants was quantified by ELISA. Untreated UM-SCC-74A cells produced very high levels of VEGF (1247 pg/ml/10^6^cells) and miR-34a transduction significantly reduced VEGF production (56%) (Figure 6C). Similarly, miR-34a transfection in UM-SCC-74B cells significantly reduced VEGF production (45%) (Figure 6F).

### miR-34a Inhibits Endothelial Cell Proliferation, Migration and Tube Formation

**Figure 7:**
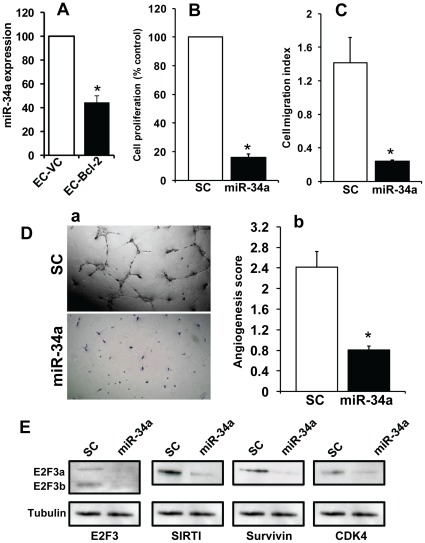
miR-34a inhibits endothelial cell proliferation, migration and tube formation. *A:* miR-34a expression in endothelial cells expressing Bcl-2 (EC-Bcl-2) or vector alone (EC-VC) was analyzed by real time RT-PCR. *B-C:* Endothelial cells (EC) transfected with 50 nM of miR-34a or scrambled control RNA (SC) were used for different assays after 48 hrs of transfection. Endothelial cell proliferation *(B)* and cell migration *(C)* was measured using Xcelligence system. *D:* Endothelial cell tube formation assay was performed on Matrigel coated lab-tech chamber slides. At the end of assay, each chamber was photographed under microscope, and the area occupied by endothelial cell tubes was calculated using NIS-Elements-BS software (Nikon) and expressed as an angiogenic score *(E)*. Whole cell lysates were Western blotted (72 hrs post transfection) and probed with specific antibodies against E2F3a/b, SIRT1, survivin and CDK4 proteins. Equal protein loading in Western blotting was verified by stripping the blots and reprobing with tubulin antibody.

In our previous study, we have shown Bcl-2 expression is significantly elevated in tumor-associated endothelial cells (EC-Bcl-2) [39], [40] and our results from this study suggest that miR-34a expression is significantly decreased in Bcl-2 expressing endothelial cells as compared to endothelial cells containing empty vector alone (EC-VC) (Figure 7A). We next examined if miR-34a could directly affect angiogenic function of endothelial cells *in vitro*. Transfection of miR-34a in endothelial cells significantly inhibited cell proliferation (85%) and migration (84%) (Figure 7B-C). Similarly, miR-34a significantly inhibited the ability of endothelial cells to form tubular structure on Matrigel (Figure 7Da-b). In addition, ectopic expression of miR-34a in endothelial cells significantly inhibited the expression of the target proteins E2F3a/b, SIRT1, survivin and CDK4 (Figure 7E).

## Discussion

Head and neck squamous cell carcinomas (HNSCC) are the most frequent malignancies of the upper aerodigestive tract [41]. In many cases, these cancers are diagnosed at a very late stage (III or IV). These cancers are known to be very severe and they can strip away a person’s voice, distort the face and rob the basic abilities to eat, drink and swallow. Although advancements in the techniques for surgery, radiation and chemotherapy have increased the local control of HNSCC, the overall survival rates have not improved significantly over the last three decades [42], [43]. The high mortality rate in these patients is in large measure due to local tissue invasion by the primary tumor as well as acquisition of resistance to chemotherapy and radiation treatment. Therefore, it is imperative that new therapeutic strategies are developed to increase the long-term survival of these patients as well as decrease the adverse effects associated with concurrent chemo-radiation regimen. In order to develop tumor specific therapies, recent research efforts have attempted to exploit the biological differences that may exist between normal and malignant cells.

Recent studies have demonstrated that microRNAs are differentially expressed in tumors as compared to normal tissues [8]. In our real-time PCR analysis of the head and neck cell lines, miR-34a expression was markedly downregulated in all the HNSCC cell lines that we tested. Similarly, Scapoli et al, have shown in their recent study that miR-34a levels are significantly downregulated in head and neck squamous cell carcinoma [44]. It is now being recognized that head and neck cancers can be subdivided into two distinct tumor types; HPV-negative and HPV-positive tumors and these tumor types have significantly different clinical outcome [45], [46]. Recently, HPV infection was shown to down-regulate miR-34a levels by destabilizing tumor suppressor p53 protein in cervical cancer [47], [48]. However, in our study, we did not observe any significant difference in miR-34a levels in HPV-positive as compared to HPV-negative head and neck cancer cell lines. In another study, Wald et al, also did not observe any significant difference in miR-34a levels in HPV-positive verses HPV-negative head and neck cancer cell lines [49]. This could be due to the fact that very few HPV positive head and neck cancer cell lines are available due to the difficulty in establishing HPV positive head and neck cell lines. In addition, the few HPV positive cell lines that are available might not represent the true nature of HPV positive head and neck tumors. We therefore selected two HNSCC cell lines (UM-SCC-74A and UM-SCC-74B) that are HPV negative and contain functional wild-type p53 for the *in vitro* and *in vivo* experiments, although they were not among the cell lines with the lowest miR-34a expression. The comparatively higher miR-34a levels in these cell lines could most likely be due to the presence of wild-type p53 in these cells as miR-34a has been shown to be a direct transcriptional target of p53 [23], [24]. UM-SCC-74B cell line is derived from an intraoral head and neck tumor and it is a relatively sensitive cell line to chemotherapy and radiation treatment. In contrast, UM-SCC-74A cell line is derived from the base of the tongue tumor and is highly resistant to both chemotherapy and radiation treatment [50].

Similar to HNSCC cell lines, miR-34a expression was also significantly downregulated in tumor samples from head and neck cancer patients. This remarkable decrease in miR-34a in head and neck tumors suggested to us that its deregulation in HNSCC may be playing a role in the progression of head and neck cancer. To test this hypothesis, we performed a series of experiment using *in vitro* as well as *in vivo* models. Our results demonstrate that ectopic expression of miR-34a strongly suppresses multiple tumorigenic functions (e.g. proliferation, and colony formation) of head and neck cancer cells. In addition, ectopic expression of miR-34a significantly inhibited cell migration even after taking into account the effect of miR-34a on cell proliferation at the same time point (60% cell migration inhibition as compared to 20% cell proliferation inhibition at 24 hrs). These tumor suppressive effects of miR-34a are mediated by changes in a number of target mRNAs including MYCN, CDK4, cyclin D1, SIRT1 and Bcl-2 [18], [51], [52], [53]. In this study, we looked at changes in protein expression of some of the known targets of mir-34a (e.g. SIRT1, CDK4, E2F3) and found them to be altered upon ectopic miR-34a expression (Figure S1). We decided to conduct an in-depth analysis of the effect of miR-34a and E2F3 in HNSCC. In this study we show that miR-34a mediates its tumor suppressor effects predominantly through E2F3a isoform, as overexpression of miR resistant E2F3a significantly rescued the effects of miR-34a on cell proliferation and colony formation. Recently, Chen et al, demonstrated that out of two different E2F3 isoforms, E2F3a showed significantly higher oncogenic potential as compared to E2F3b [54]. Similarly, Reimer et al, have shown that although both E2F3 isoforms were overexpressed in tumor samples, but only E2F3a expression directly correlated with tumor stage and residual disease in ovarian cancer patients [55]. In addition, the same group has also shown that there was a strong correlation between E2F3a expression and activated EGFR in ovarian cancer specimens and that E2F3a was a key player in EGFR driven cell proliferation [56]. The EGFR pathway is abnormally activated in HNSCC and overexpression of EGFR has been associated with poor response to chemotherapy and poor survival in head and neck cancer patients [57]. Partial rescue of miR-34a function by E2F3b in our studies could be due to some functional overlap often observed in E2F3a and E2F3b [28], [30]. Another important observation we made in this study is that ectopic expression of miR-34a in head and neck cancer cells also significantly downregulated survivin expression. Recently, miR-34a was shown to modulate survivin promoter activity [34]. However, it is not known if miR-34a-mediated regulation of survivin promoter activity is due to its direct effect or mediated via another protein. Our rescue experiments with miR resistant E2F3a suggest that miR-34a may be inhibiting survivin expression via E2F3a [35]. We also observed an inverse correlation between miR-34a and survivin expression (low miR-34a and high survivin) in most of the tumor samples from HNSCC patients. Survivin is an important oncogene and its expression is directly associated with enhanced proliferation, resistance to chemotherapy [58], [59], reduced apoptosis [60], enhanced angiogenesis [61], poor outcome [58], [62] and increased rate of tumor recurrence [63]. These results provide a novel mechanistic role for the miR-34a-E2F3a-survivin axis in mediating miR-34a tumor suppressor function.

The strong tumor suppressor effect of miR-34a on HNSCC cell lines observed during *in vitro* assays was further supported by its effects on *in vivo* xenograft tumor growth. In addition, miR-34a also significantly inhibited tumor angiogenesis by downregulating a key angiogenic factor VEGF. It has been shown that VEGF levels can be regulated by a number of miR-34a target proteins including E2F3, Myc and c-met [36], [37], [38]. Furthermore, our results demonstrate that miR-34a can also regulate tumor angiogenesis by directly inhibiting angiogenic functions of endothelial cells by downregulating a number of key proteins including E2F3, SIRT1, survivin and CDK4. Taken together, our results demonstrate an important tumor suppressor and anti-angiogenic function for miR-34a in head and neck cancers.

## Materials and Methods

### Ethics Statement

This study was approved by the institutional review board at the Ohio State University and complied with all provisions of the Declaration of Helsinki. All animal work has been conducted according to the Ohio State University IACUC Animal ethic committee and was approved by this committee (Animal Welfare Assurance Number A3261-01).

### Patient Samples, Cell Lines and Reagents

Tumor and adjacent normal tissue samples were collected from head and neck cancer patients undergoing surgical resection at the James Comprehensive Cancer Center at The Ohio State University. Use of these tissues was approved by the Ohio State University institutional review board. A board certified pathologist diagnosed all tumor tissue as HNSCC. Normal samples were collected from areas adjacent to the tumor tissue but outside the tumor margins. RNA was isolated from fresh frozen tissues samples using TRIzol reagent (Invitrogen). The isolated RNA was dissolved in RNase-free water and stored at −70°C. Out of the 15 tumor samples, 6 were from oral cavity, 5 from oropharynx and 4 from larynx. Head and neck squamous cell carcinoma (HNSCC) cell lines UM-SCC-74A, UM-SCC-74B, UM-SCC-47, UM-SCC-49, UM-SCC-38 were obtained from Dr. Thomas E. Carey (University of Michigan). UD-SCC-2 was obtained from Dr. Henning Bier (Heinrich-Heine University, Germany). CAL 27, FaDu and SCC-25 were purchased from ATCC. The identity of all the tumor cell lines was confirmed by STR genotyping (Identifiler Kit, Applied Biosystems, Carlsband, CA) and cell line characteristics are presented in Table S1. Normal human oral keratinocytes (HOK) were purchased from ScienCell (Carlsbad, CA). Human epidermal keratinocytes, adult (HEKa) and human epidermal keratinocytes, neonatal (HEKn) were purchased from Invitrogen (Carlsbad, CA). All HNSCC cell lines, except SCC-25 were cultured in DMEM supplemented with 10% fetal bovine serum containing 1% penicillin/streptomycin (Invitrogen, Carlsbad, CA) and 1% Non-essential amino acids. SCC-25 was cultured in DMEM/F12 supplemented with 10% FBS, 1% penicillin/streptomycin, 400 ng/ml hydrocortisone. HOK, HEKa and HEKn were grown in keratinocyte growth medium (Invitrogen, Carlsbad, CA). Primary human dermal microvascular endothelial cells (ECs) were purchased from Lonza (Walkersville, MD). ECs were maintained in Endothelial Cell Basal Medium-2 (EBM-2) containing 5% FBS and growth supplements. Primary antibody against E2F3a (clone 3E2F04) was purchased from Thermo Scientific (Fremont, CA), E2F3a/b (C-18) and CDK4 (C-260) from Santa Cruz (Santa Cruz, CA), CD31 (clone MEC 13.3) from Diannova (Franklin Lakes, NJ) and survivin, SIRT1 and tubulin were purchased from Cell Signaling (Danvers, MA).

### Quantitative Reverse-transcription PCR (qRT-PCR)

RNA was extracted from the HNSCC cell lines and normal keratinocytes (HOK, HEKa and HEKn) using the MirVANA kit (Ambion). TaqMan microRNA assay specific for miR-34a (Assay ID 000426) was used to detect and quantify mature miR-34a. For Survivin qRT-PCR, RNA was transcribed into cDNA and amplified with TaqMan primer/probe (Hs03043576_m1). The assays were performed in accordance with manufacturer’s instructions (Applied Biosystems, Carlsband, CA). miRNA and mRNA expression was normalized to RNU48 and OAZ1, respectively using the 2^-ΔΔCt^ method [64].

### Ectopic Expression of miR-34a in HNSCC Cells and Endothelial Cells

Precursor human miR-34a or scrambled control miRNA (Applied Biosystems) transfection in HNSCC tumor cells and EC was performed using Lipofectamine 2000 (Invitrogen) as per manufacturer’s instructions. In brief, HNSCC cells or endothelial cells were cultured in 6-well plates till they reached 60% confluence. Cells were washed and further cultured in the respective growth media minus antibiotics. In separate tubes, miR-34a (50 nM) and lipofectamine 2000 were diluted in OPTI-MEM medium and incubated for 5 minutes. After incubation, miR-34a and lipofectamine were mixed together and further incubated for 30 minutes. At the end of incubation, miR-34a and lipofectamine solution was carefully added to 6-well plates containing cells. After 24 hours, cells were washed and cultured in complete growth media containing antibiotics. To check transfection efficiency, FITC-labeled scrambled miRs were used and we consistently observed >80% transfection efficiency. Seventy two hours post transfection, cells were used for all the subsequent experiments.

### Transduction of Tumor Cells with E2F3a and E2F3b

Retroviral particles were used to overexpress miR resistant E2F3a and E2F3b [29] (a kind gift from Dr. Brian D. Dynlacht, New York University) in head and neck cancer cell lines as described previously [65]. In brief, the E2F3a and E2F3b constructs or the vector alone was introduced into PT67 amphotropic packing cells with Lipofectamine 2000. Viral supernatants were collected after 24 h, centrifuged, filtered, and stored at −80^o^C. Head and neck tumor cells (UM-SCC-74A and UM-SCC-74B) were transduced with viral supernatants supplemented with 4 µg/ml polybrene 3 times every 4 hrs. The last infection was left overnight and the next morning cells were washed and cultured in fresh medium. Stable clones overexpressing E2F3a and E2F3b were selected by treating with puromycin (2 µg/ml) for 3 days.

### Cell Proliferation

Tumor and endothelial cell proliferation were examined by MTT assay [66] (Roche Diagnostics, Indianapolis, IN) and Xcelligence system [67] using the RTCA DP instrument (Roche, Mannheim, Germany). For MTT assay, tumor or endothelial cells were plated in flat-bottomed 96-well microtitre plates at a density of 2×10^3^ and 5×10^3^ cells/well respectively. At the completion of incubation, cell proliferation was assessed by adding 10 µl of MTT labeling reagent into each well and incubating at 37°C for 4–6 hrs (4 hrs for tumor cells and 6 hrs for HDMEC). Reaction was stopped by adding solubilization solution and incubating the plates at 37°C overnight. The plates were read on a microplate reader (Sectramax 190, Molecular Devices Corp., Sunnyvale, CA) at a wavelength of 590 nm. The percentage cell proliferation for each group was calculated by adjusting the control group to 100%. For Xcelligence system proliferation assay, 100 µl of media (DMEM for tumor cells and EGM for EC) containing 2% FBS was added to the wells. After 1 hr of equilibration with media, 100 µl of cell suspension (3,000 tumor cells or 5,000 endothelial cells) was added to each well. Cell proliferation was monitored and expressed as percentage cell proliferation.

### Motility Assay

Tumor and endothelial cell motility were examined by scratch assay [66] and Xcelligence system [67]. For scratch motility assay, a fine groove was made using a sterile pipette tip in about 90% confluent cells. The migration of cells was monitored microscopically using Nikon Eclipse Ti microscope with DS-Fi1 camera. For Xcelligence system migration assay, 160 µl of media (DMEM for tumor cells and EGM for EC) containing 10% FBS was added to the lower chambers. Upper chamber (sensor surface facing down) was then carefully assembled on top of lower chamber and 50 µl of serum free media was added to the wells. After 1 hr of equilibration with media, 100 µl of cell suspension (50,000 cells/well in serum free media) was added to each well. Cell migration to lower chamber was monitored and expressed as cell migration index at 24 hrs.

### Tumor Cell Colony Formation Assay

Tumor cells (5x10^3^) were plated in 60 cm petri dishes and cultured at 37°C. After 14 days of culture, colonies were stained with crystal violet (0.005%) for 20 minutes and photographed. Alpha Innotech (San Leandro, CA) imaging software was used to quantify tumor cell colony numbers.

### ELISA

Tumor cells were cultured in 6-well plates till they are 80% confluent. Cells were washed and fresh media was added. After 24 hours, culture supernatants were collected and cell number in each well was counted. VEGF levels in culture supernatants were measured using Quantikine human ELISA kits (R&D Systems, Minneapolis, MN) as per manufacturer’s instructions and normalized to 1x10^6^ cells.

### Western Blot Analysis

Whole cell lysates were separated by 4–12% NuPAGE Bis-Tris gels (Invitrogen, Carlsbad, CA) and transferred onto PVDF membranes. Nonspecific binding was blocked by incubating the blots with 3% BSA in Tris buffered saline containing 0.1% Tween-20 (TBST) for 1 hr at room temperature (RT). The blots were then incubated with primary antibody in TBST +3% BSA at 4°C overnight. After washing with TBST, the blots were incubated with horseradish peroxidase-conjugated sheep anti-mouse IgG (1∶5,000) or with donkey anti-rabbit IgG (1∶5,000) for 1 hr at RT. An ECL-plus detection system (GE Healthcare, Piscataway, NJ) was used to detect specific protein bands. Protein loading in all the experiments was normalized by stripping the blots and then re-probing with anti-tubulin antibody. Alpha Innotech (San Leandro, CA) imaging software was used to quantify Western blot bands.

### Matrigel in vitro Endothelial Tube Formation Assay

Endothelial cell tube formation was performed on Matrigel coated chamber slides as described previously [68]. Each assay was photographed (Nikon Eclipse Ti microscope with DS-Fi1 camera) at 40x magnification and total area occupied by endothelial cell derived tubes in each chamber was calculated using software (NIS-Elements-Basic Research, Nikon, Melville, NY) and expressed as an angiogenic score.

### Xenograft Tumor Model

Tumor cells (1 x10^6^) were mixed with 100 µl of Matrigel and injected subcutaneously in the flanks of SCID mice (n = 5) as described previously [40]. Tumor volume measurements began on day 3 and continued twice a week until the end of the study. The length and width of the tumors were measured using a digital caliper and tumor volumes were calculated using the formula, volume (mm^3^) = L×W^2^/2 (length L, mm; width W, mm). After 18 days, tumors samples were carefully removed. A piece of each of the primary tumor was used to extract RNA to confirm the presence of miR-34a by real-time PCR. Rest of the tumors were fixed with 4% paraformaldehyde and then processed to form paraffin embedded tissue blocks for immunohistochemistry. Tumor sections were stained for angiogenesis (CD31) as described previously [68]. Microvessel density was calculated by counting 5 random high power fields (200x).

### Statistical Analysis

Data from all the experiments are expressed as mean ± SEM from a minimum of 3 independent experiments. The miR-34a expression in patient samples and head and neck cancer cell lines (Figure 1) was analyzed by Mann-Whitney test. To examine the inverse relationship between miR-34a and survivin expression in patient samples (Figure 4F), miR-34a levels for each tumor sample was plotted against the inverse of survivin levels and Pearson correlation coefficient was calculated. The rest of the data was statistically analyzed by two-way analysis of variance or Student’s t test (wherever applicable) and a p value of <0.05 was considered significant.

## Supporting Information

Figure S1 **miR-34a significantly downregulates SIRT1, CDK4, E2F3a/b and survivin protein expression.** UM-SCC-74A cells were transfected with miR-34a or SC. Seventy two hrs after transfection whole cell lysate was prepared and Western blotted using antibody specific for SIRT1 (top panel) or CDK4 (second panel) or E2F3a/b (third panel) or survivin (fourth panel). Equal protein loading in Western blotting was verified by stripping the blots and reprobing with tubulin antibody.
(TIF)

Figure S2 **miR-34a significantly inhibits tumor cell proliferation.** UM-SCC-74A cells were transfected with miR-34a or SC. Seventy two hrs after transfection, cells were plated in 96 well plates and cell proliferation was examined at different time points using MTT assay.
(TIF)

Table S1 **Head and neck cancer cell line characteristics.**
(TIF)
