# Supplementary figures and images for: Dysregulation of MicroRNA-34a Expression in Head and Neck Squamous Cell Carcinoma Promotes Tumor Growth and Tumor Angiogenesis
Source: PLoS One. 2012 May 22;7(5):e37601. doi: 10.1371/journal.pone.0037601 (PMC3358265; doi:10.1371/journal.pone.0037601)

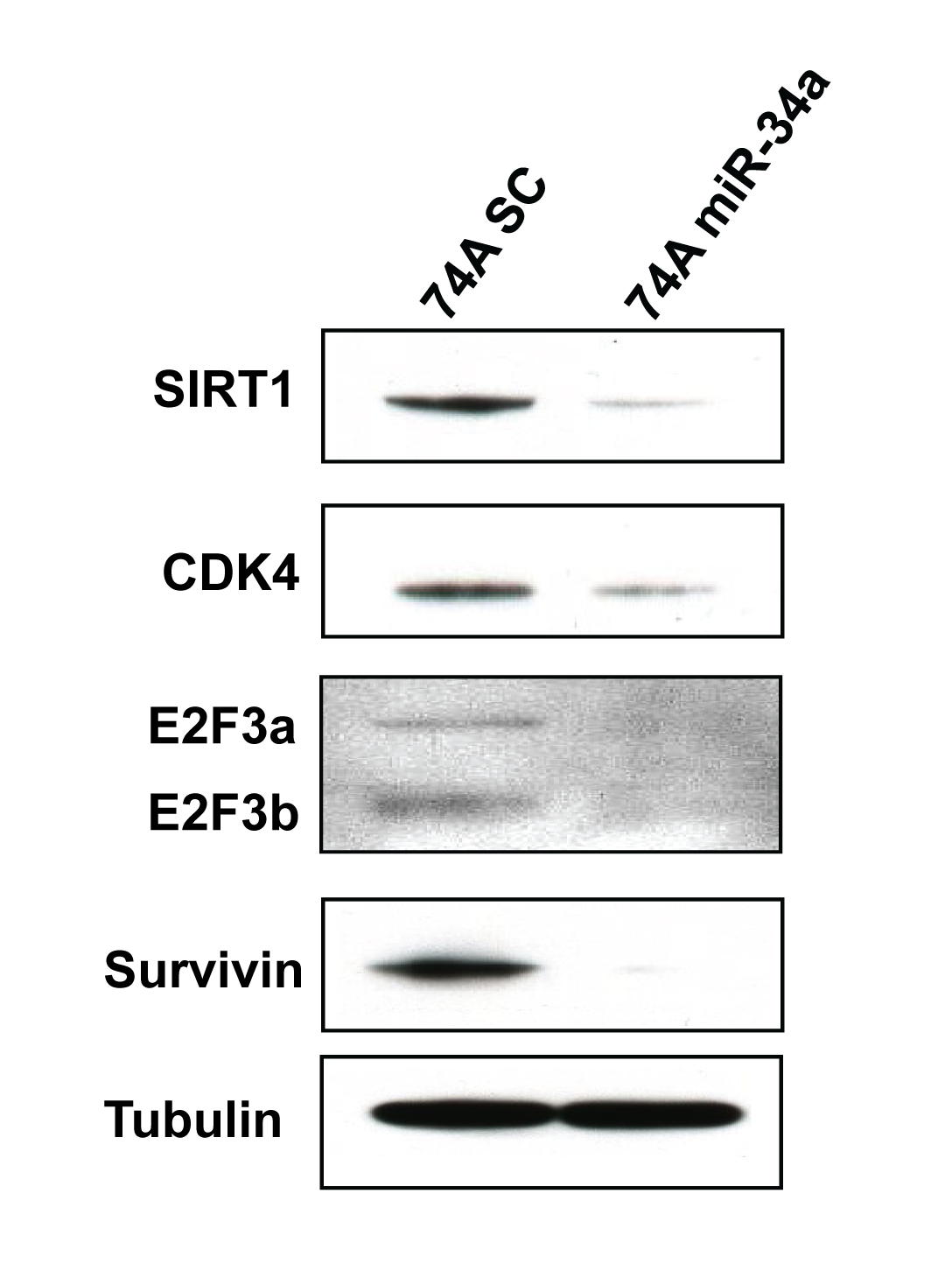

Supplement: Figure S1 — miR-34a significantly downregulates SIRT1, CDK4, E2F3a/b and survivin protein expression. UM-SCC-74A cells were transfected with miR-34a or SC. Seventy two hrs after transfection whole cell lysate was prepared and Western blotted using antibody specific for SIRT1 (top panel) or CDK4 (second panel) or E2F3a/b (third panel) or survivin (fourth panel). Equal protein loading in Western blotting was verified by stripping the blots and reprobing with tubulin antibody. (TIF) [file pone.0037601.s001.tif]

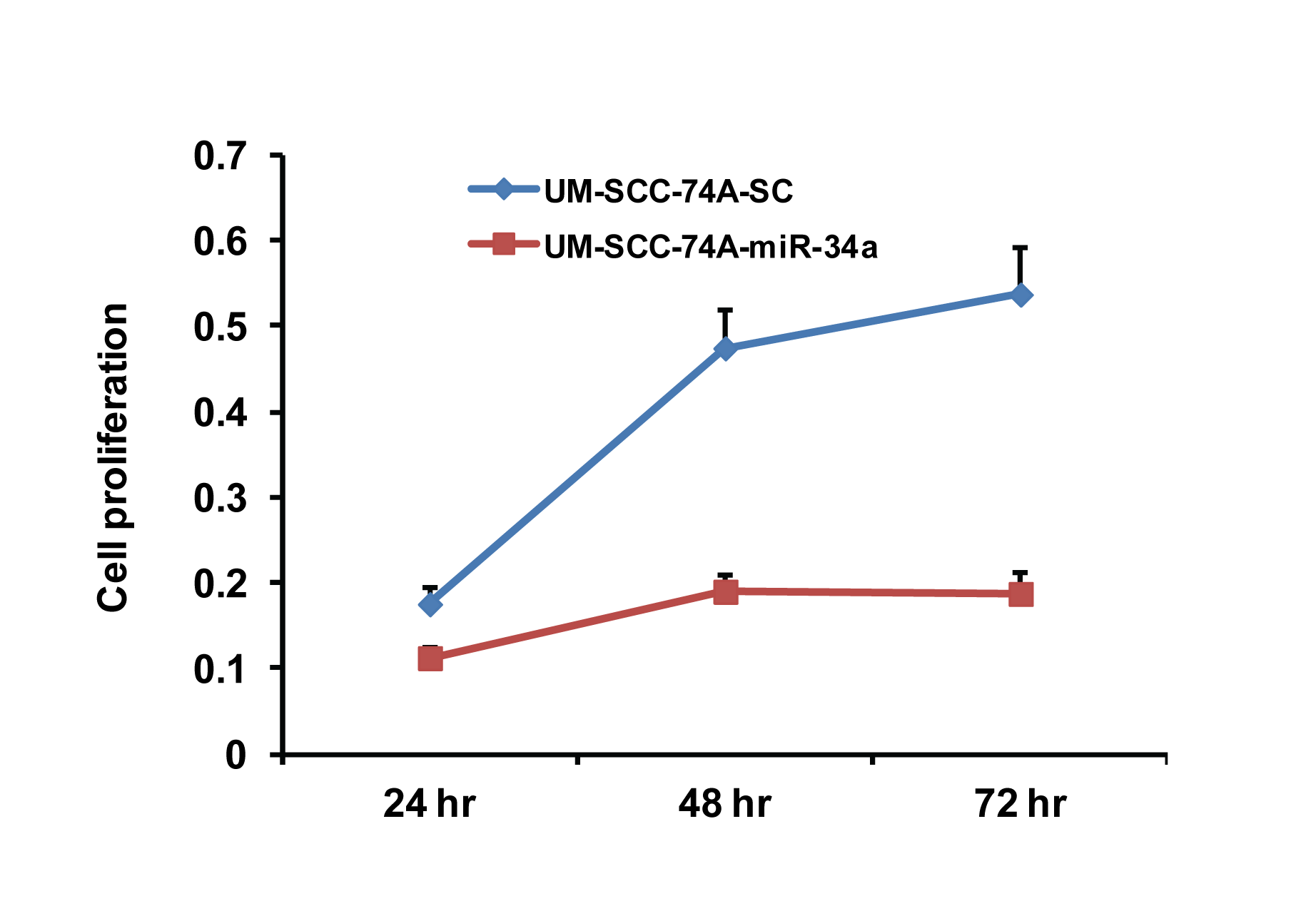

Supplement: Figure S2 — miR-34a significantly inhibits tumor cell proliferation. UM-SCC-74A cells were transfected with miR-34a or SC. Seventy two hrs after transfection, cells were plated in 96 well plates and cell proliferation was examined at different time points using MTT assay. (TIF) [file pone.0037601.s002.tif]

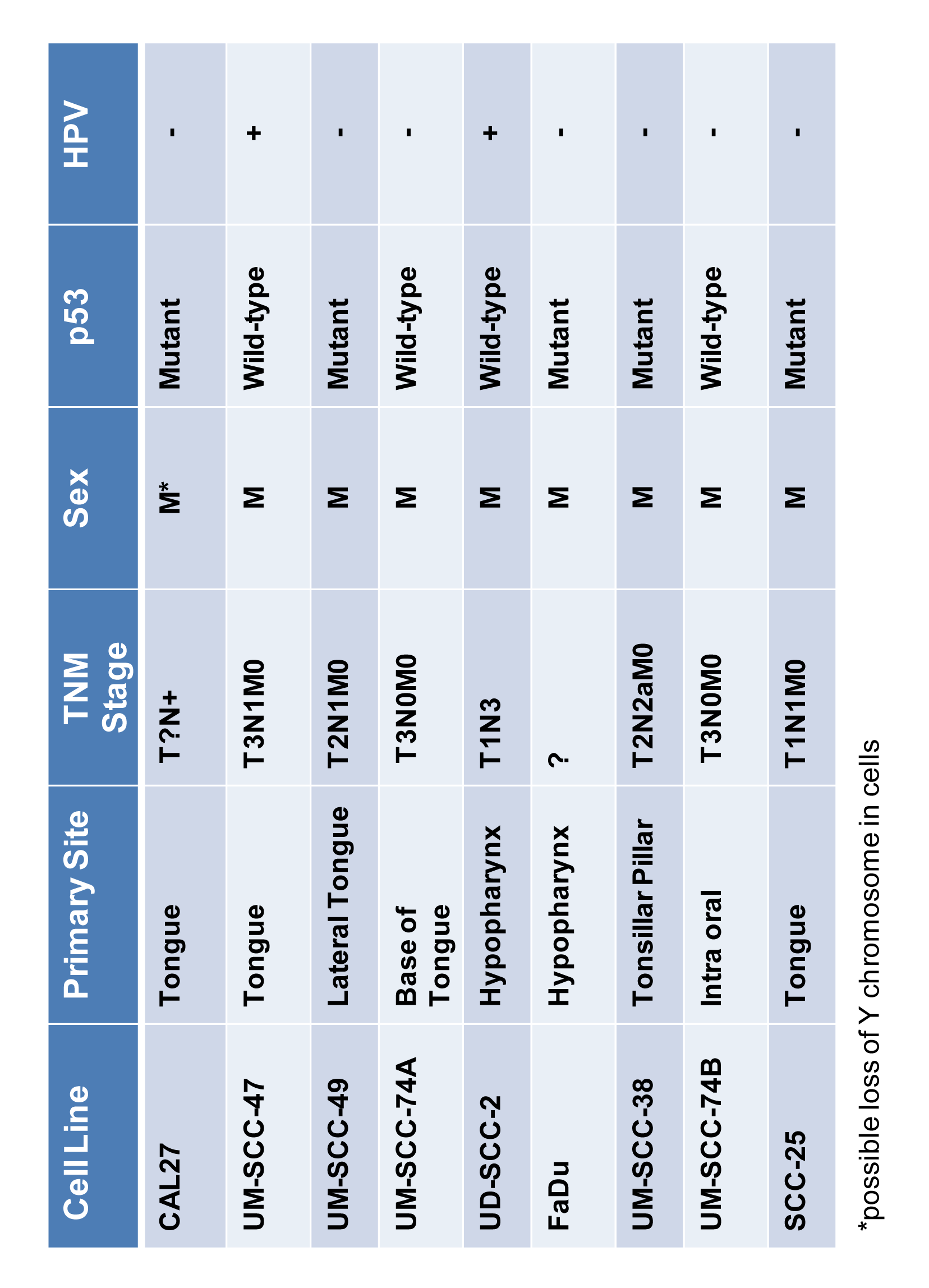

Supplement: Table S1 — Head and neck cancer cell line characteristics. (TIF) [file pone.0037601.s003.tif]
